# A Case of Leucocythæmia

**Published:** 1877-04

**Authors:** Albert E. Hoadley


### A CASE OF LEUCOCYTHÆMIA.

By DR. ALBERT E. HOADLEY.

Mrs. D. Of Irish birth; aged about 35; married.

I first saw her Dec. 17th, 1874. I was called to control nasal hemorrhage, which had been very obstinate and almost continuous for three days, and would not be controlled by ordinary means. Four physicians had been in attendance before I was called, but none of them attempted to plug the posterior nares, which treatment I resorted to and accomplished without delay or difficulty, and the bleeding stopped. From this time my patient lay in a semi-conscious condition; pulse very soft and rapid; tongue very pale, dry and clean, and the only nourishment that she could be induced to partake of was new butter-milk, which she was allowed to use in large quantities—from four to six quarts a day. At this time her death was looked for every day, and on the fourth day her condition was no more favorable than on the first day of my attendance, (the 17th); but on the 22d she was up and dressed, and by the 25th had washed nearly every washable article in the house, as they had all been soiled with blood; and on the 1st day of January, 1875, she walked more than a mile. She looked well and said that she never felt better; her appetite from the 25th was ravenous, and raw onions was her favorite article of food. The only medicines used were tr. ferri mur., alternated with Quiniæ Sulph. I lost sight of her, until April 21st, 1876, when she again sent for me. I found her bleeding from the nose as before, (she said she had been getting gradually weaker for the past six weeks when the hemorrhage again commenced); but as the bleeding was slow and only of three or four hours' duration, I endeavored to control it without resorting to the nasal plug. So I employed the nasal douche, with a very strong solution of alum, and afterwards Monsels' solution of iron by injection, and at the same time giving fl. ex. ergot with the glycerole of gallic acid; but the bleeding would not be controlled after 24 hours diligent use of these means; so the plugs were again resorted to, which was effectual, and then followed the same train of conditions, and the same treatment first described; and May 1st, 1876, my patient was again on her feet, able to walk and work. But this was of short duration, for in a few weeks she again began to decline, and on the 9th of October she again went to bed and was very low, and for four weeks was not expected to live from one day to the next. Subsequently she recovered a little strength, her digestive power improved, and she was able to be about, though quite unable to endure any considerable exertion or excitement, but this apparent improvement was of brief duration.

She again went to bed on the 22d of Dec., 1876, and sank rapidly; on the 25th, she had a large passage of blood from the bowels—at least a quart—and on the next day a similar passage, but less in quantity. There was no positive pain, but a sense of fullness in the region of the spleen and over the stomach, with marked tenderness on pressure. She also had an uncomfortable sensation about the heart, with an anæmic murmur. From the time of the second passage of blood from the bowels, she sank rapidly, suffering greatly from dyspnœa, and died on the 29th of Dec., retaining her mind clear to the last.

The post mortem, made twenty hours after death, showed the lungs, the entire alimentary canal and the genital organs to be in healthy condition. The heart was soft and considerably dilated, with its muscular tissue thoroughly infiltrated with fat. The liver was at least one-third larger than normal, and there was also fatty degeneration well marked; the clear fat globules could readily be discerned with the naked eye.

The spleen was enlarged to three or four times its normal size, and on section presented an intensely engorged condition,—almost black in color,—and there oozed from the cut surface a dark, dirty-looking, bloody serum. The kidneys were very nearly normal in size, but on longitudinal section the cortical substance that approached in color and appearance to health was about one-eighth of an inch thick; the rest of the kidney was as yellow as fat could make it, and the pelvis of the kidney was the seat of several little masses of fat, each the size of very small peas. Upon the exterior of the body there was a fair amount of adipose tissue; it was fully one-fourth of an inch thick upon the abdomen.

Dr. Danforth will append the microscopy of this case.

*Microscopy.*—The white blood corpuscles were largely in excess; many of the red corpuscles were shriveled, crenated, or otherwise deformed. The muscular tissue of the heart had undergone fatty degeneration, so that in some places the sarcolemma seemed to be filled with granular debris; other fibres were in the incipiency of fatty degeneration—that is, in the stage of so-called "cloudy swelling," while a few could be seen which were scarcely affected. The fatty change was evidently the result of a process of slow starvation, since there was no organic structural lesion of its valves, or of the great vessels. The tubuli of both kidneys were very extensively degenerated, the epithelial cells being loaded with fat and granular matter. I have no doubt that a more complete microscopic examination of the tissues of the body would have developed quite generally the degenerative changes which usually follow prolonged mal-nutrition. I made several ante mortem examinations of the patient's urine, and on each occasion found albumen abundant, and a profuse deposit of granular casts and fat-bearing epithelium. It may, perhaps, be fairly questioned which was the primary disease, leucocythæmia or morbus Brightii; but the question cannot now be authoritatively answered.

